# Head and Neck Characteristics as Risk Factors For and Protective Factors Against Mild Traumatic Brain Injury in Military and Sporting Populations: A Systematic Review

**DOI:** 10.1007/s40279-022-01683-2

**Published:** 2022-05-06

**Authors:** Nicholas J. Cooney, Paul Sowman, Nathan Schilaty, Nathaniel Bates, Timothy E. Hewett, Tim L. A. Doyle

**Affiliations:** 1 School of Psychological Sciences, Faculty of Medicine, Health and Human Sciences, Macquarie University, Sydney, NSW Australia; 2 Department of Neurosurgery and Brain Repair, University of South Florida, Tampa, FL USA; 3 Center for Neuromusculoskeletal Research, University of South Florida, Tampa, FL USA; 4 Department of Orthopaedics, The Ohio State University Wexner Medical Center, Columbus, OH USA; 5 Hewett Global Consulting, Minneapolis, MN USA; 6 Rocky Mountain Consortium for Sports Injury Research, Aspen, CO USA; 7 Department of Health Sciences, Faculty of Medicine, Health and Human Sciences, Macquarie University, Sydney, NSW Australia

## Abstract

**Background:**

Investigators have proposed that various physical head and neck characteristics, such as neck strength and head and neck size, are associated with protection from mild traumatic brain injury (mTBI/concussion).

**Objectives:**

To systematically review the literature and investigate potential relationships between physical head and neck characteristics and mTBI risk in athletic and military populations.

**Methods:**

A comprehensive search of seven databases was conducted: MEDLINE, EMBASE, CINAHL, Scopus, SPORTDiscus, Cochrane Library, and Web of Science. Potential studies were systematically screened and reviewed. Studies on military and athletic cohorts were included if they assessed the relationship between physical head-neck characteristics and mTBI risk or proxy risk measures such as head impact kinematics.

**Results:**

The systematic search yielded a total of 11,723 original records. From these, 22 studies met our inclusion criteria (10 longitudinal, 12 cross-sectional). Relevant to our PECO (Population, Exposure, Comparator, and Outcomes) question, exposures included mTBI incidence and head impact kinematics (acceleration, velocity, displacement) for impacts during sport play and training and in controlled laboratory conditions. Outcome characteristics included head and neck size (circumference, mass, length, ratios between these measures), neck strength and endurance, and rate of force development of neck muscles.

**Discussion:**

We found mixed evidence for head and neck characteristics acting as risk factors for and protective factors against mTBI and increased susceptibility to head impacts. Head-neck strength and size variables were at times associated with protection against mTBI incidence and reduced impact kinematics (14/22 studies found one or more head-neck variable to be associated with protection); however, some studies did not find these relationships (8/22 studies found no significant associations or relationships). Interestingly, two studies found stronger and larger athletes were more at risk of sustaining high impacts during sport. Strength and size metrics may have some predictive power, but impact mitigation seems to be influenced by many other variables, such as behaviour, sex, and impact anticipation. A meta-analysis could not be performed due to heterogeneity in study design and reporting.

**Conclusion:**

There is mixed evidence in the literature for the protective capacity of head and neck characteristics. We suggest field-based mTBI research in the future should include more dynamic anthropometric metrics, such as neck stiffness and response to perturbation. In addition, laboratory-based mTBI studies should aim to standardise design and reporting to help further uncover these complicated relationships.

## Key Points

**Table:** 

This article provides a comprehensive review of the relationship between physical head and neck characteristics and mild traumatic brain injury (mTBI) risk
There was mixed evidence in the literature for the protective capacities of head and neck characteristics against mTBI
While strength and size metrics of the head and neck may have some predictive power, head impact mitigation and mTBI incidence seem to be influenced by many other variables, such as behaviour, sex, and impact anticipation

## Introduction

Concussion, commonly referred to as mild traumatic brain injury (mTBI), is a prevalent injury in sport and military settings. mTBI is caused by biomechanical forces acting on the brain, either from a direct blow to the head or elsewhere on the body. The primary cause of mTBI is inertial forces, namely linear and rotational acceleration of the brain upon impact [1]. Those afflicted with mTBI experience transient loss of normal brain function. Typically, athletes recover to pre-injury function within 2–14 days [2]. However, for 10–30% of those affected, symptoms can persist for months [3]. Experience of long-term somatic, psychological, emotional, and cognitive symptoms after mTBI is known as *post-concussion syndrome* (PCS) [4].

Several physical characteristics of the head and neck have been researched with regard to the potential role they play in mTBI incidence. Perhaps most saliently, neck strength is hypothesised as a protective factor against mTBI, especially in a sporting context. It is thought that tensing of cervical musculature increases effective movable mass, allowing those with stronger necks to more effectively distribute potentially injurious forces from impacts as compared to those with weaker necks [5]. Rate of force development of neck muscles has also been measured by some groups [6, 7], with the idea that being able to quickly reach a high level of muscle activity is important for resisting deformation after head impacts [7]. Low neck endurance has also been proposed as a risk factor for mTBI, as it is speculated that the neck dysfunction associated with reduced levels of neck endurance may in turn reduce one’s ability to react, resist forces, and stabilise the head during impact [8]. Neck size and strength differences between sexes have been hypothesised as major underlying factors that account for the higher incidence of mTBI in females [9]. The posited relationship between head and neck characteristics and mTBI has seen groups investigate how these characteristics relate to mTBI incidence. Collins et al. [10] found a significant relationship between neck strength and size in prevention of mTBI in high school athletes, showing one-pound increments in neck strength decrease the odds of mTBI by 5%. Baker et al. [8] investigated cervical muscle endurance and did not find increases to endurance times to be significantly associated with mTBI in university athletes. Other head/neck characteristics have been related to mTBI through the head-response to impacts and perturbations, and observing how these characteristics affect the injury mechanism. Alsalaheen et al. [11] performed a perturbation experiment on recreationally active adults and discovered that men and women employed different stabilisation strategies. They hypothesise that women may rely on greater neuromuscular activation to account for lower size and strength compared to males. Dezman et al. [12] found that imbalance in neck musculature symmetry in the sagittal plane led to higher head accelerations in football/soccer heading. Mihalik et al. [5] found there was no effect of cervical strength on head acceleration in youth ice hockey head impacts. Schmidt et al. [7] investigated head impact biomechanics in American football and found that athletes with higher cervical stiffness had reduced odds of sustaining higher magnitude head impacts as compared with athletes with lower cervical stiffness, and that cervical strength and size had no significant effect in mitigation of head impact severity. Importantly, some of these characteristics are modifiable and therefore head/neck training programs may offer protection against sports-related and military mTBI.

Research into these head/neck characteristics broadly falls into two categories: studies performed in controlled laboratory settings and those in live field-based sporting situations. The former is useful as it allows experimenters to keep variables consistent across subjects, but applicability to the real world is not as obvious as in the latter. In laboratory-based settings, impacts are understandably kept to sub-concussive levels. Mechanisms of imparting these impacts on participants include football/soccer heading [12–18], perturbation through a weight dropped via a pulley system [6, 7, 9, 11, 19] or by an impact sled [20, 21]. Field-based settings have the advantage of being able to ethically observe the effect of head/neck characteristics on impacts over the mTBI threshold, as well as on mTBI incidence itself. These studies often record head/neck outcomes pre-season and then track mTBI incidence [8, 10, 22] or head impact kinematics (HIK) [5, 7, 23] over one or more seasons. Schmidt et al. [7] recorded both laboratory- and field-based data, which may provide insight into how measurements and outcomes relate between these two settings.

In the current literature, the role of head and neck size, neck strength (maximum strength, rate of strength development, and endurance), and neck stiffness in prevention of mTBI is inconclusive. This systematic review aims to determine the relationships between these characteristics of the head and neck with mTBI incidence and injury risk. Understanding these relationships will help elucidate major risk factors and help to inform decision making for training and prevention of mTBI.

## Methods

This systematic review was registered with Open Science Framework (OSF) Registries on 24 November 2021 (https://osf.io/f6gv8). The Preferred Reporting Items for Systematic Reviews and Meta-Analyses (PRISMA) guidelines were followed when conducting and reporting this review [24]. The PRISMA guidelines contain a checklist and flow-chart that indicate the items to include within a systematic literature review and the phases in which to conduct the review.

### Search Strategy and Eligibility Criteria

A systematic search of the literature was conducted using seven databases from their date of inception up until 8 November 2021. The databases used were MEDLINE, EMBASE, CINAHL, Scopus, SPORTDiscus, Cochrane Library and Web of Science. Our review question was defined within the PECO (Population, Exposure, Comparator, and Outcomes) framework. Keywords to describe the population (athletes and military), exposure (mTBI or proxy measures of mTBI risk), and outcome (head and neck characteristics) were used in the search strategy (see Table 1). The review was not aimed at specific comparisons between concussions and interventions; so as not to limit the papers that were captured, comparator keywords were not used in the search strategy. The lower cut-off of 13 years of age allowed for studies on teenagers to be included. As 65 years is a common age of retirement, the upper cut-off ensures a full occupational cohort was captured. The final search term was formed by concatenation of the terms as such: [population terms] AND [exposure terms] AND [outcome terms]. Keywords targeted the title and abstract section of potential records in the databases. Corresponding subject headings were also included when they existed in each database (i.e., subject heading “Brain Concussion/” in Medline). We also performed a manual search of the included studies’ reference lists.

**Table 1:** List of keywords used in database searches broken down into PECO (Population, Exposure, Comparator, and Outcomes) format

PECO element	Keywords used
Population	(sport* OR athlet* OR rugby OR basketball OR football OR hockey OR lacrosse OR soccer OR wrestling OR equestrian OR "martial art*" OR boxing OR "physically active") OR (military OR veteran* OR soldier* OR army OR navy OR “air force” OR “armed force*” OR “special force*” OR marines)
Exposure	("mild traumatic brain injur*" OR mtbi OR concuss*) OR (rotation OR acceleration OR kinematic* OR biomechanic*)
Outcome	neck OR cervical OR head

Articles were included that: (1) took a biomechanical, performance, anthropometric, clinical, or other physical recording of the head and/or neck, (2) determined the likelihood of exposure to mTBI, either through direct incidence or proxy measures of mTBI risk (increased head acceleration during perturbation/impact, reduced ability for dynamic stabilisation, etc.), (3) observed an adult military population in active duty or training, or a 13- to 65-year-old sporting population, of any sport, at any level of competition, (4) presented original data, and (5) were published in English in a peer-reviewed scientific journal. Articles were excluded if they: (1) observed mTBI occurrence in civilian vehicle accidents (as opposed to military blast-related vehicle accidents), (2) observed mTBI occurrence as a result of a fall, (3) observed populations of children (0–12 years old) and older adults (65 + years), or where results from 13- to 65-year-old participants were not reported separately to children and older adults, (4) only reported physical measures that are not directly focused on the head and/or neck (postural sway, balance, etc.), (5) only reported measures of brain and genetic structure at the micro-level (gene studies, brain protein biomarkers, imaging studies, etc.), (6) only reported on neurocognitive performance measures, (7) studied an animal population, (8) used simulations or models instead of human participants, (9) were unavailable as full-text documents, and (10) were reviews, conference abstracts, case reports, commentaries, or letters to the editor. When there was uncertainty about whether a record met the inclusion criteria, it was kept for further assessment in the full text screening stage. No studies were excluded based on quality assessment.

### Quality Assessment

Studies that met the inclusion criteria were reviewed by one author (NJC), and a second author (TLAD, PS) then independently reviewed each study that met the inclusion criteria using the Mixed Methods Appraisal Tool (MMAT) [25]. The screening and quantitative non-randomized criteria were applied to each of the included studies. Conflicts between author ratings were discussed with an independent third author until an agreement was reached.

### Data Extraction

Data were extracted into a spreadsheet by one author (NJC) and reviewed by all authors independently. Data extracted included study details (author, year, setting), participant characteristics (number, age, sex, sport code and level of play for athletes, branch of military for military studies), aims, methodology, exposures, outcomes, and general findings.

### Data Synthesis and Analysis

A meta-analysis was planned, but during data extraction it became clear there was a large amount of heterogeneity within the included studies. As such, a statistically supported meta-analysis could not be performed. Results are therefore reported in tabular and narrative format.

## Results

### Study Selection and Quality Assessment

**Fig. 1:**
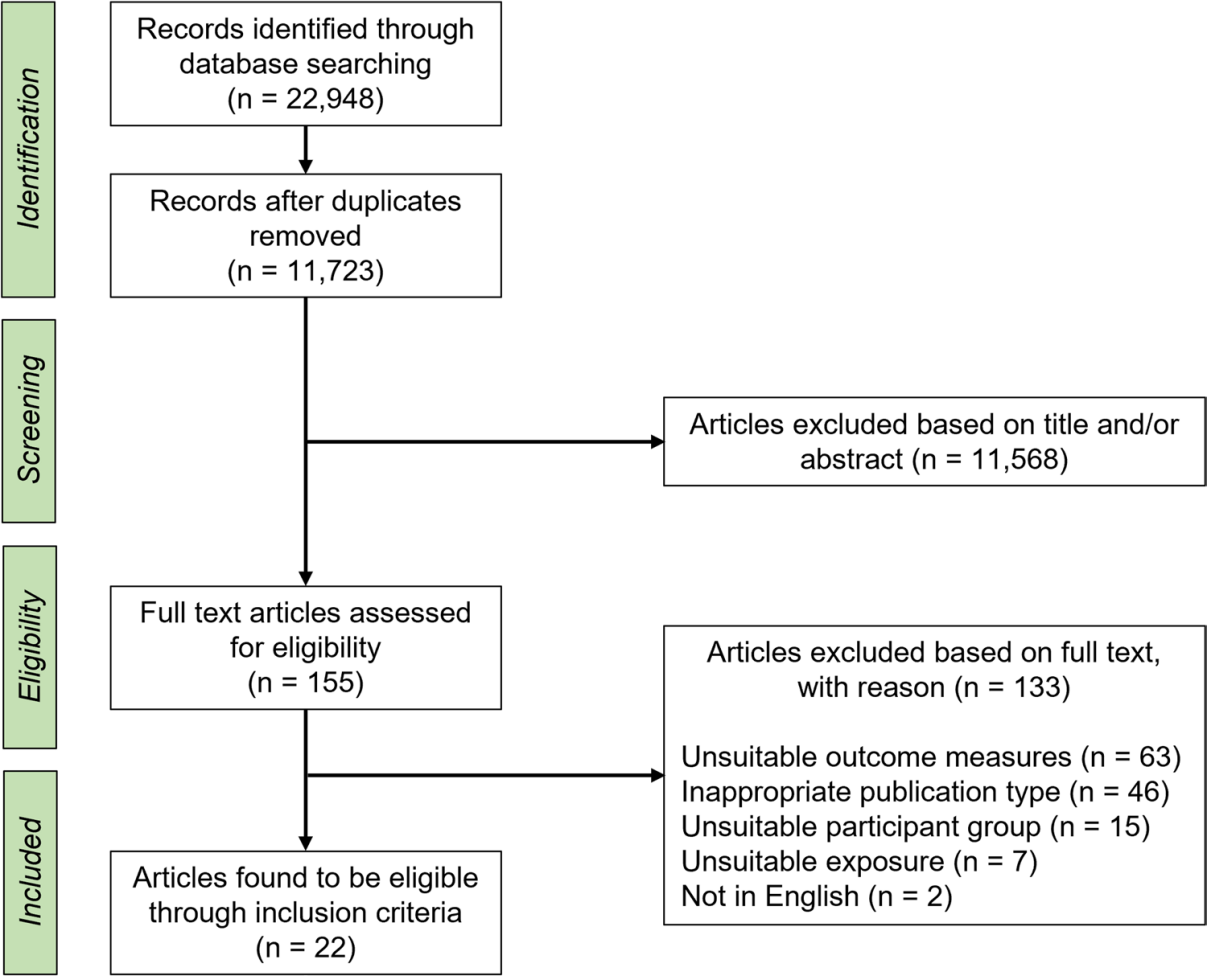
PRISMA flowchart showing the stages and number of records in the systematic review

The results of the search are shown in Fig. 1. Inclusion and exclusion criteria were used on title and abstract for 11,723 records. Out of 155 articles screened in the full text stage, 22 fully met our inclusion criteria. Reasons for exclusion at this stage are given in the PRISMA flowchart (Fig. 1).

Table 2 reports the methodological quality assessment of the included studies. All studies fulfilled both screening questions of the MMAT (“Are there clear research questions?” and “Do the collected data allow addressing the research questions?”). Twenty of the 22 included studies met all criteria of the MMAT. The average score was 4.9/5. One study [21] did not meet the criteria on item 3.3 (“Are there complete outcome data?”) and another study [14] did not meet the criteria on item 3.4 (“Are the confounders accounted for in the design and analysis?”).

**Table 2:** Methodological quality assessment as rated using the Mixed Methods Appraisal Tool (MMAT) [25]

	Screening questions		Methodological quality criteria (quantitative nonrandomized)					
Study	S1. Are there clear research questions?	S2. Do the collected data allow the research questions to be addressed?	3.1. Are the participants representative of the target population?	3.2. Are measurements appropriate regarding both the outcome and intervention (or exposure)?	3.3. Are there complete outcome data?	3.4. Are the confounders accounted for in the design and analysis?	3.5. During the study period, is the intervention administered (or exposure occurred) as intended?	Total Methodological Quality Score
Alsalaheen et al. [11]	Y	Y	Y	Y	Y	Y	Y	5/5
Baker et al. [8]	Y	Y	Y	Y	Y	Y	Y	5/5
Becker et al. [13]	Y	Y	Y	Y	Y	Y	Y	5/5
Bretzin et al. [14]	Y	Y	Y	Y	Y	N	Y	4/5
Caccese et al. [15]	Y	Y	Y	Y	Y	Y	Y	5/5
Collins et al. [10]	Y	Y	Y	Y	Y	Y	Y	5/5
Debison-Larabie et al. [19]	Y	Y	Y	Y	Y	Y	Y	5/5
Dezman et al. [12]	Y	Y	Y	Y	Y	Y	Y	5/5
Eckner et al. [6]	Y	Y	Y	Y	Y	Y	Y	5/5
Esopenko et al. [22]	Y	Y	Y	Y	Y	Y	Y	5/5
Fitzpatrick et al. [26]	Y	Y	Y	Y	Y	Y	Y	5/5
Gutierrez et al. [16]	Y	Y	Y	Y	Y	Y	Y	5/5
Kelshaw et al. [23]	Y	Y	Y	Y	Y	Y	Y	5/5
Mawn et al. [20]	Y	Y	Y	Y	Y	Y	Y	5/5
Mihalik et al. [5]	Y	Y	Y	Y	Y	Y	Y	5/5
Morris and Popper [21]	Y	Y	Y	Y	N	Y	Y	4/5
Müller and Zentgraf [27]	Y	Y	Y	Y	Y	Y	Y	5/5
Schmidt et al. [7]	Y	Y	Y	Y	Y	Y	Y	5/5
Teymouri et al. [17]	Y	Y	Y	Y	Y	Y	Y	5/5
Tierney et al. [9]	Y	Y	Y	Y	Y	Y	Y	5/5
Tierney et al. [18]	Y	Y	Y	Y	Y	Y	Y	5/5
Williams et al. [28]	Y	Y	Y	Y	Y	Y	Y	5/5

*Y* Yes, *N* No

Table 3 summarises the 22 articles that met inclusion criteria. The articles are separated into two distinct groups: (1) field-based concussion and/or HIK studies, and (2) laboratory-based perturbation studies. The key difference between the groups is that Group 1 recorded data from real-world, live sporting settings, while Group 2 used controlled settings in which study participation was the only task. The study by Schmidt et al. [7] is the only study that falls into both groups. The results of our included articles are presented in these groups.

**Table 3:** Characteristics and findings of included articles (relative to our PECO (Population, Exposure, Comparator, and Outcomes) and inclusion criteria)

Study	Study design	Description	Population	Exposure	Outcomes	General finding
*Group 1: Field-Based Concussion and/or Head Impact Kinematics Studies (n* = *8)*						
Baker et al. [8]	Longitudinal	Athlete neck endurance was measured pre-season. Over a single season, upon sustaining a concussion, athletes were re-tested immediately and throughout recovery	130 (68 F) university varsity athletes (ice hockey, football/soccer, basketball). Age not reported	1) An in-season mTBI. 12 (6 M) athletes sustained a mTBI (9.2% of study population)	1) Neck endurance	The authors used two-sample Wilcoxon rank sum tests to compare endurance scores of athletes who sustained a mTBI with those who did not. Endurance times were organised and tested by 1) continuous variables of absolute time, 2) dichotomous variables separated by the population mean time, and 3) interval variables where times were divided into six groups. Athletes who sustained a mTBI had lower average neck endurance than those who did not, however the result did not reach significance (*p* = 0.55). No significance was found when comparing scores from dichotomous (*p* = 0.49) or interval variables (*p* = 0.50), or when comparing absolute endurance times of males (*p* = 0.55) and females (*p* = 0.84) independently
Collins et al. [10]	Longitudinal	Head and neck anthropometrics were recorded for high school athletes prior to sports seasons across two years. mTBI incidence was recorded throughout the season	6,662 high school athletes (football/soccer, basketball, lacrosse)	1) mTBI reported by athletic trainers. 179 (107 F) athletes sustained a mTBI (2.7% of study population)	1) Head girth 2) Neck length 3) Neck girth 4) Neck MVIC strength (flexion, extension, left and right lateral flexions)	For the overall study population, using a two-sample t-test the authors found neck girth, neck:head girth ratio, and all measures of neck strength were significantly lower (*p* ≤ 0.001) in mTBI athletes than in uninjured athletes. Neck length and the ratio between neck length and head girth were not significantly different between injured and uninjured athletes. The authors found that for every one pound increase in neck strength, odds of mTBI decreased by 5%
Esopenko et al. [22]	Longitudinal	Neck circumference was measured for incoming college athletes. Sport mTBI during college was recorded for these athletes	324 (165 F) division I collegiate athletes from 16 sports. Mean age of 18.3 ± 1.0 years	1) Sports mTBI during college athletic career. 13 (9 M) athletes sustained a mTBI (4% of study population)	1) Neck circumference 2) Proportional neck circumference (BMI/neck circumference)	Univariate analysis of variance (ANOVA) and analysis of covariance were performed with raw and proportional neck circumference as dependent variables. When comparing athletes who did versus did not sustain a mTBI, no significant differences were found for raw (*p* = 0.235) or proportional (*p* = 0.613) neck circumference
Fitzpatrick et al. [26]	Longitudinal	Blind football/soccer players wore a head-mounted accelerometer during 16 training sessions and 4 competition matches. Neck strength was measured at the mid-point of the data collection period	7 male members of the England Blind Football Squad. Mean age of 28.6 ± 9.7 years	For game- and training-related impacts (*n* = 374): 1) Peak linear head acceleration 2) Peak rotational head velocity	1) Neck MVIC strength (flexion, extension, left and right lateral flexions and rotations)	No association was found between mean neck strength with either linear acceleration (*p* = 0.685) or rotational velocity (*p* = 0.798). The authors then analysed results for each player in each direction (i.e., mean HIK for impacts on the front of the head with neck extension strength). When the data were analysed in this way using linear regression, a statistically significant relationship was found between neck strength and linear acceleration (*p* = .020) but not neck strength and rotational velocity (*p* = 0.861)
Kelshaw et al. [23]	Longitudinal	Lacrosse players wore an accelerometer and gyroscope instrumented helmet across a season of 12 games. Impacts of 20 G or higher were recorded and confirmed on video	15 male high school varsity lacrosse players. Mean age of 16.5 ± 1.3 years	For game-related, video-confirmed impacts of ≥ 20 G (*n* = 367): 1) Peak linear head acceleration 2) Peak rotational head acceleration	1) Neck MVIC strength normalised to body mass (flexion, extension, left and right lateral flexions) 2) Head circumference 3) Neck circumference 4) Neck length	The authors performed a multivariate ANOVA to investigate HIK differences between neck strength tertile groups (weak, moderate, strong). No significant differences in either linear acceleration or rotational velocity were found between the groups (*p* > 0.05). The only significant finding was correlation of neck circumference with neck extension strength (*r* = 0.63, *p* = 0.02)
Mihalik et al. [5]	Longitudinal	Youth ice hockey athletes wore accelerometer instrumented helmets during training sessions (99) and games (98). Head impacts over 10 G were recorded and analysed	37 male youth ice hockey players. Mean age of 15.0 ± 1.0 years	For game- and training-related impacts of ≥ 10 G (*n* = 7,770): 1) Peak linear head acceleration 2) Peak rotational head acceleration 3) HITsp (Head Impact Telemetry severity profile)	1) Neck MVIC strength normalised to body mass of five muscle groups (anterior cervical, anterolateral cervical, cervical rotator, posterolateral extensor, and upper trapezius). Strength was averaged between bilateral measurements where appropriate	The authors used random intercept general mixed linear models for each HIK measure across each neck strength measure. Neck strength tertile groups (weak, moderate, strong) were used in the analysis and had significantly different strength in each direction (*p* > 0.05). The only significant difference found between groups was in HITsp for upper trapezius muscle strength, however this was a positive correlation of strength and HITsp (stronger players experienced larger impacts than weaker players). No other significant relationships were found between neck strength measures and HIK between the groups
Williams et al. [28]	Longitudinal	Instrumented mouthguards were worn by rugby athletes during 13 (six male, seven female) competitive matches. Head impacts over 10 G were recorded, verified using video and waveform-analysis, and evaluated	53 (28 M) first XV university rugby union players. Mean age of 20.7 ± 1.8 years	For game-related impacts of > 10 G (*n* = 218): 1) Peak linear head acceleration 2) Peak rotational head acceleration	1) Neck MVIC strength (flexion, extension, left and right lateral flexions) 2) Head circumference 3) Neck circumference	Using independent samples t-tests, the authors found females had significantly lower neck circumference, head circumference, and neck strength (in all directions) than males (*p* < 0.001 for all measures). Using independent samples Mann–Whitney U tests, the authors found no significant differences between females and males in linear (*p* = 0.23) or rotational (*p* = 0.76) head acceleration. Interestingly, uncontrolled whiplash action occurred just once in all the impacts sustained by male athletes but was present in over half of all impacts for females
*Group 1 & 2: Studies That are Both Field- and Laboratory-Based (n* = *1)*						
Schmidt et al. [7]	Longitudinal	American football players had pre-season head/neck anthropometrics and strength measured, including their response to a perturbation protocol. Head impacts over 10 G were recorded during training and play over a single season	49 male high school (age 16.6 ± 0.9 years) and collegiate (age 20.5 ± 1.4 years) American football players	Group 1, for game- and training-related impacts of ≥ 10 G (*n* = 19,775): 1) Peak linear head acceleration 2) HITsp Group 2: 1) Angular displacement 2) Neck stiffness	1) Head circumference 2) Neck circumference 3) Head-neck segment length 4) Neck MVIC strength (flexion, extension, left and right lateral flexions) 5) Rate of neck force development (in the same directions) 6) Neck muscle physiological cross-sectional area (SCM, upper trapezius, semispinalis capitis)	The authors used random-intercept generalized logit models to calculate the odds ratio (OR) and 95% confidence interval (95% CI) of sustaining severe (> 106G) and moderate (66-106G) head impacts against mild head impacts (10-66G) for high and low performers of each outcome variable. The authors found that players with greater neck stiffness had reduced odds of sustaining severe (OR, 0.64; 95% CI, 0.46:0.89) and moderate (OR, 0.77; 95% CI, 0.61:0.96) head impacts compared to players with less neck stiffness, as measured by HITsp. Neck size and strength did not mitigate head impact severity. In fact, linemen in the high performance groups for lateral and overall neck strength had increased odds of suffering moderate as opposed to mild linear head impacts (OR, 1.78; 95% CI, 1.01:3.16; for overall neck strength). Additionally, players who developed extension torque faster had higher odds (OR, 2.10; 95% CI 1.08:4.05) of sustaining severe versus mild linear head impacts
*Group 2: Laboratory-Based Perturbation Studies (n* = *15)*						
Alsalaheen et al. [11]	Cross-sectional	Athletes undertook a controlled forced extension perturbation protocol with anticipation and preloading conditions	34 (20 M) recreationally active (8 h per week sports or exercise) adult athletes. Mean age of 23.0 ± 2.3 years	1) Peak angular head velocity 2) Extension excursion	1) Neck girth 2) SCM physiological cross-sectional area 3) Neck MVIC strength (flexion)	Using independent samples t-tests, the authors found males had significantly higher neck girth, SCM physiological cross-sectional area and neck flexion strength than females (all *p* < 0.001). Despite this, a mixed model ANOVA showed peak angular head velocity and extension excursion were not significantly different between males and females during head perturbation. The authors argue that males and females used different strategies to resist perturbation, as females demonstrated significantly higher levels of both baseline (*p* = 0.003) and peak (*p* = 0.01) electromyography response
Becker et al. [13]	Longitudinal	Football/soccer players were divided into three groups: two received a neck training intervention and the other was a control group. Athletes performed five heading conditions (standing, jumping, running, post-fatigue jumping and running) on a stationary ball before and after the intervention	33 male football/soccer players. Mean age of 20.3 ± 3.6 years	1) Peak linear head acceleration	1) Neck MVIC (flexion and extension)	The authors used a repeated measures ANOVA to analyse pre-post-effects and group differences. Neck flexion strength significantly increased for all groups post-intervention (*p* < 0.001), but no significant differences were seen between intervention and control groups (*p* = 0.055). Neck extension strength changes post-intervention were not significant (*p* = 0.216) and not significantly different between groups (*p* = 0.121). A one-way ANOVA showed that training induced alterations in peak linear head acceleration during football/soccer heading were not significant for any of the five heading conditions (*p* ≥ 0.295)
Bretzin et al. [14]	Cross-sectional	Football/soccer players performed headers at two ball speeds (11.2 m/s and 17.9 m/s)	13 (8 F) NCAA Division I football/soccer athletes. Mean age of 19.8 ± 0.9 years	1) Peak linear head acceleration 2) Peak rotational head velocity	1) Head-neck segment length 2) Head-neck segment mass 3) Neck girth 4) Neck MVIC strength (flexion, extension, left and right lateral flexions and rotations)	Using independent samples t-tests, the authors showed males to have significantly stronger necks than females in flexion (*p* = 0.012) and left lateral flexion (*p* = 0.002). Males had higher strength values in the other four directions, but these results did not reach significance (*p* ≥ 0.075). Males experienced lower levels of linear head acceleration and rotational head velocity compared to females for both heading speeds, but only the differences in rotational head velocity reached significance. Using Pearson correlations, the authors found neck girth had negative correlations with rotational velocity and linear accelerations at low ball speeds, but only with linear acceleration at high ball speeds. Linear acceleration also correlated negatively with 3/6 (flexion, left lateral flexion, left rotation) and 4/6 (flexion, left and right lateral flexions, left rotation) muscle groups for 11.2 m/s and 17.9 m/s ball speeds respectively
Caccese et al. [15]	Cross-sectional	Football/soccer players completed standing, forward headers at ball speed 11.2 m/s	100 (58 F) football/soccer players. Mean age of 17.1 ± 3.5 years. 26 (18 F) participants were youth whose results are not included for our purposes	1) Peak linear head acceleration 2) Peak rotational head acceleration	1) Head width 2) Head length 3) Head depth 4) Head girth	Statistical analysis using a multivariate ANOVA by the authors included the youth group, whereby a significant difference was seen between males and females for linear and rotational head acceleration. This interaction effect was not seen in a multivariate sex by age analysis. Head circumference showed the same trend—males had significantly larger heads than females when considering all ages groups, but no sex by age interaction effect was seen
Debison-Larabie et al. [19]	Cross-sectional	Ice hockey players undertook a controlled perturbation protocol in four directions (neck flexion, extension, left and right lateral flexion) either with or without knowledge of force application	16 (8 M) varsity and competitive league ice hockey players. Female and male mean age of 20.6 ± 1.3 years and 22.1 ± 1.6 years, respectively	1) Peak angular head acceleration	1) Head:neck girth ratio 2) Neck volume	Using independent samples t-tests, the authors found males had significantly larger neck volume (*p* = 0.01) and significantly lower head:neck girth ratio (*p* = 0.01) than females. The authors used four separate 2-way mixed ANOVA to test for significant acceleration differences between sexes in each perturbation direction. Males had lower head acceleration than females for all perturbation directions except extension, but this trend only reached significance for flexion (*p* = 0.01) and left lateral flexion (*p* = 0.05)
Dezman et al. [12]	Cross-sectional	Football/soccer players performed forward headers at a mean ball speed of 4.3 m/s ± 0.7 m/s	16 (8 M) division I and II collegiate football/soccer players. Mean age of 20.5 ± 1.9 years	1) Peak translational head acceleration 2) Peak rotational head acceleration	1) Neck MVIC strength (flexion, extension) 2) Neck flexion–extension strength difference	Independent samples t-tests found that all neck strength measures (flexion, extension, flexion–extension difference) and HIK (translational and rotational head acceleration) were not significantly different between males and females. The authors then pooled the male and female data and computed Spearman rho correlations between neck strength measures and HIK. Correlations between mean neck strength measures and HIK variables were insignificant (i.e., neck flexion/extension strength were not correlated with either type of head acceleration, *p* > 0.05). A significant correlation between neck strength imbalance (flexion–extension difference) and angular head acceleration was found (rho = 0.497; *p* = 0.05). The correlation between neck strength imbalance and linear acceleration also trended towards significance (rho = 0.485; *p* = 0.057)
Eckner et al. [6]	Cross-sectional	Contact sport athletes underwent a controlled perturbation protocol in four directions (flexion, extension, right lateral flexion or left axial rotation)	46 (24 M) athletes from a range of contact sports. Female and male mean age of 15.0 ± 4.4 years and 16.3 ± 5.0 years, respectively. 26 (14 M) of these participants were in a youth group and not included for our purposes	1) Peak linear head velocity 2) Peak angular head velocity Note: both normalised for impulsive load differences (divided by impulse potential energy)	1) Neck circumference 2) SCM physiological cross-sectional area 3) Neck MVIC strength (flexion, extension, left lateral flexion, and right axial rotation) 4) Rate of neck force development (in the same directions)	Statistical analysis using multivariate linear mixed models included youth participants, whereby neck strength and size had significant main effects for rotational and linear velocity after perturbation (all *p* < 0.001). When adjusting for sex and age in the models these effects remained significant. The same trend is seen for the older athletes (comparing college age and older males and females), but the authors did not report statistical analysis for this sub-group as it was not the aim of their study
Gutierrez et al. [16]	Cross-sectional	Football/soccer players performed 15 standing headers on throw-ins aiming left, right, and forward (5 in each direction)	17 female varsity high school football/soccer players. Mean age of 15.9 ± 0.9 years	1) Peak linear head acceleration	1) Neck MVIC strength (flexion, extension, left and right lateral flexions)	The authors computed Pearson correlations between peak linear head acceleration during heading and neck strength in all four directions. They reported moderate and consistent negative correlations for neck strength in all directions with head acceleration for all heading directions (*r* = − 0.500:– 0.757, all *p* < .05)
Mawn et al. [20]	Cross-sectional	Navy personnel had linear and angular head acceleration measured during impact sled runs of approximately 10 G for forced neck flexion	15 US Navy enlisted men. Age not reported	1) Peak linear head acceleration (for x- and z-axes of the sled) 2) Peak angular head acceleration (around the y-axis of the head)	1) Head mass (estimated) 2) Neck circumference 3) Neck length	The authors used regression models to see if head/neck anthropometrics could predict the three head acceleration variables measured. Anthropometrics included the three measured head/neck outcomes as well as “stockiness quotient” (neck circumference:neck length ratio), head mass:stockiness quotient ratio, and head mass:neck circumference ratio. They found a strong relationship between anthropometry and linear z-axis acceleration (up-down, R^2^ = 0.75, significance level = 99.9), and moderate relationships between anthropometry and linear x-axis (forwards-backwards, R^2^ = 0.38, significance level = 93.3) and angular y-axis (sagittal plane, R^2^ = 0.44, significance level = 97.5) accelerations
Morris and Popper [21]	Cross-sectional	Volunteers underwent two perturbation protocols on an impact sled, 6.5 G for forced neck flexion and 4 G for right lateral flexion	34 (18 M) US Air Force members. Age not reported	1) Mean corrected mouth deflection (head displacement/ neck length)	1) Neck MVIC strength (extension and left lateral flexion) 2) Headrest force resultant (extension and left lateral flexion)	The authors found a positive correlation between mean corrected mouth deflection and headrest force resultant for both males (*r* = − 0.93) and females (*r* = − 0.88). Static neck strength did not predict head motion. They argue subjects were not motivated under static strength testing, but under correct conditions (during sled impact) participants maximally activate neck musculature and this force negatively correlates with head motion
Müller and Zentgraf [27]	Longitudinal	Football/soccer players were divided into an intervention group and a control group. The intervention group completed a strengthening protocol in addition to regular football/soccer training, while the control group participated in regular training only. The intervention group performed 12 purposeful standing headers pre- and post-intervention. Ball speed was low (9.4 m/s) or high (10.8 m/s). Only males headed the ball at the higher speed	37 (24 M) football/soccer players aged 15–18 years old	1) Peak linear head acceleration	1) Neck MVIC strength (flexion, extension, left and right lateral flexions) 2) Neck MVIC strength symmetry (lateral only) 3) Functional neck strength (flexion and extension) 4) Functional neck endurance 5) Neck length 6) Neck circumference 7) Neck volume	Through computation of multiple linear regressions the authors found that for low ball velocity, neck volume and functional neck flexion strength were the only significant anthropometric predictors of head acceleration. Similarly, for high ball velocity, functional neck flexion strength was the only significant anthropometric predictor. Regarding their strengthening intervention, repeated measures multivariate ANOVAs revealed significant differences in neck strength outcomes between the control and intervention groups. Univariate tests showed the strengthening intervention had a significant beneficial effect on all strength variables measured except frontal plane neck strength symmetry. A significant decrease in peak linear head accelerations (-1.5 g; 95% CI -2.6:-0.4; *p* = 0.009) during heading between pre- and post-intervention measurements for low but not high ball velocity was seen
Teymouri et al. [17]	Cross-sectional	Football/soccer players defended three free kicks with a header. Mean speed of free kicks in the study was 18.0 ± 2.4 m/s	16 football/soccer players (sex not reported). Mean age of 17.5 ± 1.9 years	1) Head speed (before and after heading) 2) Head momentum (before and after heading) 3) Force exerted by the ball on the head	1) Neck MVIC strength (flexion) 2) Head circumference	The authors used Pearson correlations to test for significant relationships between force exerted on the participants’ head from the ball during heading and their other measured variables. No correlation was found between neck flexion strength and force exerted by the ball on the head (*p* = 0.51). A significant negative correlation (*r* = − 0.50, *p* = 0.00) was found between this force and head circumference (participants with larger heads experienced less force from the ball)
Tierney et al. [9]	Cross-sectional	Participants underwent forced flexion and extension perturbation protocols either with or without knowledge of force application	40 (20 M) physically active participants (30 min exercise five or more times a week). Mean age of males and females was 26.3 ± 4.3 years and 24.2 ± 4.1 years, respectively	1) Peak angular head acceleration 2) Angular displacement 3) Neck stiffness	1) Head-neck length 2) Head-neck mass 3) Neck girth 4) Neck MVIC strength (flexor and extensor)	Using t-tests, the authors found males had significantly larger head-neck mass and neck girth (both *p* = 0.000), but not head-neck length (*p* = 0.453), than females. From multivariate ANOVAs and follow-up ANOVAs, females demonstrated significantly lower neck strength (49% less) and stiffness (29% less) than males. Females had significantly higher levels of peak angular head acceleration (50% more) and angular displacement (39% more) than males during perturbations
Tierney et al. [18]	Cross-sectional	Football/soccer players performed 12 standing, forward headers. Four headers were completed for three headgear conditions (2 types of headgear and without headgear)	44 (29 F) football/soccer players. Mean age of males and females was 20.3 ± 2.9 years and 19.5 ± 1.8 years, respectively	1) Peak linear head acceleration 2) HIC	1) Head-neck segment length 2) Head-neck segment mass 3) Neck girth 4) Neck MVIC strength (flexion, extension)	From independent samples t-tests, females demonstrated significantly lower head-neck segment mass (*p* < 0.001) and length (*p* = 0.004), neck girth (*p* < 0.001), and neck flexion and extension strength (both *p* < 0.001) than males. From post hoc independent samples t-tests, females showed significantly greater linear head acceleration for both headgear conditions (*p* ≤ 0.006), but not in the control condition (*p* = 0.164), than males. No significant differences were seen between the sexes for HIC in any of the three headgear conditions. Pearson correlations were computed between head-neck variables and peak linear head acceleration during heading. Without headgear, head-neck mass significantly and negatively correlated with resultant head acceleration (*r* = − 0.327, *p* ≤ 0.05). With either type of headgear, all head/neck outcomes correlated significantly and negatively with resultant head acceleration (*r* = − 0.101: − 0.576, *p* ≤ 0.05)

*95% CI* 95% confidence interval, *ANOVA* analysis of variance, *F* female, *G* acceleration of gravity (9.8 m/s^2^), *HIC* head injury criteria, *HITsp* Head Impact Technology severity profile, *HIK* head impact kinematics, *M* male, *mTBI* mild traumatic brain injury, *MVIC* maximum voluntary isometric contraction, *NCAA* National Collegiate Athletic Association, *OR* odds ratio, *SCM* sternocleidomastoid

### Group 1: Field-Based Concussion and/or Head Impact Kinematics Studies (*n* = 8)

Studies in Group 1 used field-based measurements of concussion and/or head kinematics and correlated them with head and neck characteristics [5, 7, 8, 10, 22, 23, 26, 28]. The relationship between each variable and mTBI is reported in Table 3.

#### Exposures

Baker et al. [8] followed 130 university varsity athletes (ice hockey, football/soccer, and basketball) throughout their respective seasons. They reported 12 participants (six male) sustained a mTBI in their study (9.2% of study population). Collins et al. [10] followed 6662 high school athletes (lacrosse, football/soccer, and basketball) throughout the 2010 and 2011 academic years. They reported 179 participants (72 male) sustained a mTBI (2.7% of study population). Esopenko et al. [22] followed 324 National Collegiate Athletic Association (NCAA) Division I athletes over their whole college career in 16 sports. They reported 13 participants (nine male) sustained a mTBI (4% of study population). The other five studies in this group related HIK resulting from training and/or game-related head impacts to head-neck characteristics [5, 7, 23, 26, 28].

#### Measurement of Head Impact Kinematics

All five of the HIK studies in Group 1 reported peak linear head acceleration [5, 7, 23, 26, 28], three reported peak rotational head acceleration [5, 23, 28], Fitzpatrick et al. [26] reported peak rotational head velocity, and Mihalik et al. [5] and Schmidt et al. [7] also reported Head Impact Technology severity profile (HITsp), which is a weighted composite score that takes into account rotational and linear acceleration as well as impact location and duration [7]. Three articles [5, 7, 28] only included impacts over 10 G, while Kelshaw et al. [23] had a threshold of 20 G. Fitzpatrick et al. [26] did not report an impact threshold. Kelshaw et al. [23] and Williams et al. [28] both used video to confirm impacts, and Williams et al. [28] additionally used waveform-analysis to rule out head acceleration data from non-impact events (biting, shouting, and insertion or removal of instrumented mouthguard).

#### Measurement of Neck Strength

Neck strength in flexion, extension, and right and left lateral flexion was reported by Collins et al. [10] (in pounds), Fitzpatrick et al. [26] (in Newtons), Kelshaw et al. [23] (unitless and normalised with participant body mass), Schmidt et al. [7] (as peak torque and rate of torque development), and Williams et al. [28] (in Newtons). Fitzpatrick et al. [26] also measured left and right rotational neck strength. Baker et al. [8] were alone in investigating neck endurance as measured by the deep neck flexor endurance test (DNFET). Three of the studies analysed their results based on groups. Kelshaw et al. [23] and Mihalik et al. [5] investigated head kinematic outcomes based on grouping into neck strength tertiles for each direction of neck strength measured, while Schmidt et al. [7] investigated their head kinematic results based on high and low performers (two groups, median-split) for each head/neck outcome.

#### Additional Outcome Measures

Head and neck anthropometrics reported in the six studies included head circumference [7, 10, 23, 28], neck circumference [7, 10, 22, 28], proportional neck circumference [22], neck length [10, 23], and head-neck segment length [7]. Schmidt et al. [7] also investigated neck muscle size as well as neck stiffness assessed through perturbation, which are both mentioned in more detail in Sect. 3.3.1.

#### Exposure-Outcome Relationships

Of the Group 1 studies that report mTBI incidence [8, 10, 22], only one found significant differences in head-neck outcomes for athletes who did versus did not sustain an mTBI [10]. Collins et al. [10] found neck girth, neck:head girth ratio, and all measures of neck strength were significantly lower in athletes who sustained an mTBI than those who did not. Esopenko et al. [22] found no significant differences in raw or proportional neck circumference measures in athletes who sustained an mTBI compared to those who did not. Baker et al. [8] found that athletes who sustained an mTBI had lower average neck endurance than those who did not; however, the difference was not significant between groups.

For the Group 1 studies that reported HIK [5, 7, 23, 26, 28], four found no significant impact mitigation effect associated with stronger neck muscles [5, 7, 23, 28]. In fact, two found that athletes with stronger necks were at times more likely to sustain more severe head impacts than those with weaker necks [5, 7]. Initially, Fitzpatrick et al. [26] similarly found no association between neck strength and a reduction in HIK. However, upon analysing their data directionally (i.e., mean HIK for impacts on the front of the head correlated with neck extension strength), they found that neck strength significantly and negatively associated with linear acceleration but not rotational velocity in this way. Two studies included head-neck size variables in their analyses with HIK, and neither found significant impact mitigation effects associated with any measured size variables [7, 28]. Schmidt et al. [7] found that players with stiffer necks had reduced odds of sustaining severe and moderate head impacts compared to players with less neck stiffness, as measured by HITsp.

### Group 2: Laboratory-Based Perturbation Studies (*n* = 15)

Studies in Group 2 used controlled, laboratory-based methods of perturbation of the head and correlated head kinematic response with head and neck characteristics. Methods used to perturb the head were a load attached via pulley system [6, 7, 9, 11, 19], football/soccer heading [12–18, 27], or an impact sled [20, 21]. The relationship between each variable and mTBI is reported in Table 3.

#### Load-Drop Studies (*n* = 5)

Five studies utilized head load applicators that employed headgear attached to a load via a pulley system and imparted a force on the head when the load was dropped [6, 7, 9, 11, 19].

*Measurement of head kinematics *To capture motion during perturbations, four studies used camera-based motion capture systems, 3D [11, 19], 2D [9] or infrared [6]. Schmidt et al. [7] used an electromagnetic motion capture system. Motion during perturbation was reported in terms of peak angular acceleration [9, 19], peak angular velocity [6, 11], peak linear velocity [6], and angular displacement [7, 9, 11].

*Types of perturbation *Loading conditions differed between studies; three studies varied force based on participant body mass [6, 7, 11] while the other two did not vary the load between participants [9, 19]. All five papers assessed forced neck extension, while four also examined forced flexion [6, 7, 9, 19]. Eckner et al. [6] also assessed right lateral flexion and left axial rotation, and Debison-Larabie et al. [19] left and right lateral flexion. A “pre-load” was used in two studies, where a load smaller than the dropped load was supported by the participant before the perturbation trial occurred [7, 11]. Alsalaheen et al. [11] also performed perturbations without pre-load. All five studies subjected participants to perturbation while relaxing neck musculature. Two studies asked participants to brace for the impact by pre-activating (tensing) neck musculature [6, 9]. Four studies had a condition where participants were given an indication (i.e., a countdown) of when the weight would be dropped [7, 9, 11, 19]. Eckner et al. [6] did not report whether participants had knowledge of weight drop timing.

*Measurement of neck strength *Neck strength was measured during maximum voluntary isometric contraction (MVIC) for neck flexion [6, 9, 11], extension [6, 7, 9], lateral flexion [6, 7], and right axial rotation [6]. Two studies also reported rate of neck force development [6, 7] for the same directions as their neck MVIC measures.

*Additional outcome measures *Other head and neck characteristics reported were neck stiffness [7, 9], head-neck segment length [7, 9], head-neck mass [9], head circumference [7], neck circumference/girth [6, 7, 9, 11], head:neck circumference ratio [19], neck volume [19], physiological cross-sectional area (PCSA) of the sternocleidomastoid [6, 7, 11], upper trapezius, and semispinalis capitis [7].

*Exposure-outcome relationships *Three groups compared males with females in their analyses [9, 11, 19]. Compared to females, males in these studies had significantly higher levels of neck strength, neck girth, sternocleidomastoid PCSA, neck volume, head:neck girth ratio, head-neck mass, but not head-neck length. Tierney et al. [9] found females experienced significantly greater levels of angular head acceleration and displacement than males, suggesting a beneficial effect in stabilisation of the stronger and larger head-neck segment in males. Similarly, Debison-Larabie et al. [19] found males experienced less angular head acceleration during perturbation than females, but only in flexion and left lateral flexion directions (no significant differences in acceleration were found between males and females for forced extension or right lateral flexion). Alsalaheen et al. [11], however, found no significant differences between males and females in angular head velocity or displacement during perturbation. The study by Eckner et al. [6] included youth athletes below 13 years old in their analyses, whereby neck strength and size had significant main effects for rotational and linear velocity after perturbation. When adjusting for sex and age in the models these effects remained significant. The same trend was seen for the older athletes (comparing college age and older males and females), but the authors did not report statistical analysis for this sub-group as it was not the aim of their study. Schmidt et al. [7, 9, 11] did not analyse the effects of head-neck strength and size variables on response to perturbation as this was not the aim of their study.

#### Football/Soccer Heading Studies (*n* = 8)

All eight of the studies in group 2 used controlled football/soccer heading trials to perturb the head [12–18, 27].

*Measurement of head impact kinematics *Six of the eight studies recorded HIK using accelerometers [13–16, 18, 27], with Caccese et al. [15] also utilising gyroscope data. Camera-based motion capture systems were used by four groups, 3D systems by three [12, 15, 27], and a 2D system by Teymouri et al. [17]. Teymouri et al. [17] also used a pressure gauge attached to participants’ foreheads to measure impact force of the ball on the head during trials. Six studies reported peak linear head acceleration during headers [13–16, 18, 27].^1^ Two reported peak rotational head acceleration [12, 15]. Bretzin et al. [14] reported peak rotational velocity.^2^ Tierney et al. [18] also reported Head Injury Criteria. Teymouri et al. [17] reported head speed and momentum before and after heading.

*Types of headers *Becker et al. [13] used five heading conditions in their study: standing, running, jumping, and post-fatigue running and jumping. Four of the studies used only the standing header condition [15, 16, 18, 27], with Müller and Zentgraf [27] allowing for one sidestep to complete the header. Teymouri et al. [17] used a less controlled method, instructing participants to defend a free kick from within the penalty box with a header (mean speed of 18.0 ± 2.4 m/s). They reported standing, jumping, and jumping forward headers. Two studies did not report the motion of the participant during the header [12, 14]. Four studies used ball machines to serve the ball to participants, Tierney et al. [18] used a speed setting of 9.8 m/s (22 mph), Müller and Zentgraf [27] used speeds of 9.4 m/s and 10.8 m/s (only male participants received balls at 10.8 m/s), while Bretzin et al. [14] and Caccese et al. [15] both used 11.2 m/s (25 mph) with Bretzin additionally using 17.9 m/s (40 mph). Three studies used another human to serve the ball to the participant. Dezman et al. [12] had an investigator serve the ball to participants at a mean speed of 4.3 ± 0.7 m/s. Gutierrez et al. [16] had a trained football/soccer player perform a throw-in to the participant and did not report ball speed. Becker et al. [13] were the only group to use a stationary ball. Six studies had participants head the ball forwards, either towards a target [15, 18, 27], back to the server [12, 16], or horizontally as hard as possible [13]. Gutierrez et al. [16] also had participants head the ball left and right. Participants in the study by Teymouri et al. [17] were instructed to head the ball away from the goal. Bretzin et al. [14] did not report on the direction of headers.

*Measurement of neck strength *Bretzin et al. [14] reported neck strength in six directions: flexion, extension, left and right lateral flexion, left and right rotation. Gutierrez et al. [16] and Müller and Zentgraf [27] reported neck strength in four directions: flexion, extension, left and right lateral flexion. Müller and Zentgraf [27] also reported lateral neck strength symmetry and functional neck strength and endurance (for the flexor and extensor muscle chains, these functional measurements had participants activating their neck and other muscles in functionally relevant positions for sport). Three groups reported neck flexion and extension strength [12, 13, 18]. Teymouri et al. [17] reported only neck flexion. Caccese et al. [15] were the only group not to report a measure of neck strength.

*Additional outcome measures *Five studies also included measures of head-neck size and mass. Bretzin et al. [14] and Tierney et al. [18] reported head-neck segment length and mass, and neck girth. Caccese et al. [15] reported head width, length, depth and girth. Müller and Zentgraf [27] recorded neck length and circumference and reported neck volume. Teymouri et al. [17] reported head girth as well as correlation coefficients for their head-neck anthropometric measures with force exerted from the ball onto the head.

*Exposure-outcome relationships *The majority of included football/soccer heading studies found that athletes with stronger necks and larger head-neck segments in some way demonstrated reduced HIK during heading. Gutierrez et al. [16] found moderate and consistent negative correlations between neck strength and head acceleration during heading in all directions. The males in the study by Bretzin et al. [14] had stronger necks than females (only significantly different for flexion and left lateral flexion) and experienced lower levels of linear head acceleration and rotational head velocity compared to females for both heading speeds (only the differences in rotational head velocity reached significance). They also found neck girth had negative correlations with rotational velocity and linear accelerations at low ball speeds, but only with linear acceleration at high ball speeds. Females in the study by Tierney et al. [18] had significantly lower head-neck segment mass and length, neck girth, and neck strength than males, and showed significantly greater linear head acceleration in headgear conditions but not in the control condition without headgear. Teymouri et al. [17] found that athletes with larger head circumference experienced less force from the ball during heading than those with smaller head circumference. Initially, Dezman et al. [12] found that neck flexion/extension strength were not correlated with linear or rotational head acceleration. However, they demonstrated that neck strength imbalance (flexion–extension difference) did significantly negatively correlate with angular (but not linear) head acceleration. Two heading studies included a strengthening intervention [13, 27]. Müller and Zentgraf [27] found significant beneficial effect from the intervention on neck strength and functional neck strength and endurance. Participants showed a significant decrease in peak linear head accelerations during heading between pre- and post-intervention measurements for low (9.4 m/s) but not high (10.8 m/s) ball velocity. The strengthening intervention performed by Becker et al. [13] did not produce significant strength changes between intervention and control groups (flexion strength increased for all groups, extension strength did not). Subsequently, training-induced alterations in peak linear head acceleration during football/soccer heading were not significant for any of the five heading conditions. The study by Caccese et al. [15] included youth athletes below 13 years old in their analyses, whereby a significant difference was seen between males and females in head circumference and linear and rotational head acceleration during heading (males had larger heads and experienced lower levels of HIK than females). However, multivariate sex by age analyses did not show these same interaction effects.

#### Impact Sled Studies (*n* = 2)

The final two studies in this group used an impact sled to impart force on the heads of military cohorts [20, 21].

*Measurement of head kinematics *Morris and Popper [21] measured head kinematics using infrared sensors and accelerometers. Mawn et al. [20] did not report how they measured head kinematics. Mawn et al. [20] reported linear x (forwards-backwards) and z (up-down) accelerations and angular y (in the sagittal plane) accelerations. Morris and Popper [21] reported head acceleration in terms of “mean corrected mouth deflection”, which is a unitless value calculated by dividing head displacement by neck length.

*Types of sled run *Mawn et al. [20] examined acceleration in the -Gx direction (forced flexion, or “eyeballs out”), which was approximately 10 G (10.1 ± 0.2 G). Morris and Popper [21] used a 6.5 G acceleration in the -Gx direction and a 4 G acceleration in the + Gy direction (right lateral flexion, or “eyeballs right”).

*Head-neck outcome measures *For physical head and neck characteristics, Mawn et al. [20] reported neck length, circumference, and estimated head mass, as well as ratios between these variables ( “stockiness quotient” or neck circumference:neck length ratio, head mass:stockiness quotient ratio, and head mass:neck circumference ratio), while Morris and Popper [21] reported neck extension strength during a static condition and during the sled-run (as headrest force resultant).

*Exposure-outcome relationships *Both impact sled studies found links between head-neck characteristics and resultant head motion during impact. Mawn et al. [20] performed linear regressions using the abovementioned head-neck size variables and found these variables had a distinct influence on linear and angular head acceleration. They found a strong relationship between anthropometry and linear z-axis (up-down) acceleration, and moderate relationships between anthropometry and linear x-axis (forwards-backwards) and angular y-axis (sagittal plane) accelerations. Morris and Popper [21] initially found static neck strength not to predict head motion as measured by mean corrected mouth deflection. However, they reported that headrest force resultant and static neck strength measurements did not correlate well. When using headrest force resultant as the independent variable instead of static neck strength, they were able to establish strong correlations with head motion for males and females.

## Discussion

This systematic review provides a comprehensive overview of the relationship between physical head and neck characteristics and mTBI risk. Mixed evidence was found regarding the protective capacities of head-neck strength and size variables against mTBI and head impacts. Fourteen of the 22 included studies established at least one significant association between a head-neck strength or size variable and mTBI risk reduction, while eight of the 22 studies found no significant results.

### Comparison Between Field- and Laboratory-Based Studies

Concerning how impact mitigation translates between less severe laboratory-based impacts to in-game impacts of 10 G + , the study by Schmidt et al. [7] can potentially provide insight given that they assessed both measures. Although they found no impact mitigation effect from neck strength or size, they found that increased neck stiffness and reduced displacement during a low-level (3.5% body mass dropped from 15 cm) laboratory-based perturbation both reduced the odds of sustaining higher magnitude head impacts during play compared to players who demonstrated lower stiffness and larger head displacement. Their results indicate that ability to mitigate low-level impacts may translate to high-level impacts, which justifies our inclusion of proxy mTBI risk measures in the review. Schmidt et al. [7] were not concerned with how head-neck characteristics affected head acceleration during the controlled perturbations, but other groups examined this directly. Tierney et al. [9] found that lower levels of head and neck size and neck strength in females were associated with reduced stiffness and higher head acceleration and angular displacement as compared to males. From their results, for low G impacts there is a link between head-neck strength and size to increased stiffness and reduced head acceleration and head displacement. This finding may indicate that a similar relationship might exist at high G impacts. While Schmidt et al. [7] found that increased stiffness provided a mitigative effect in high G impacts, they did not see the same effect for head-neck size or strength. This result could stem from sex differences in dynamic response, as Tierney et al. [9] compared males and females while Schmidt et al. [7] only included male participants.

### Neck Strength in Field-Based Studies

Many of the studies included in this systematic review showed varying levels of evidence that neck strength provides some protective effect against mTBI incidence and impact severity. Perhaps the most meaningful evidence is from the study by Collins et al. [10]. Their study had a large sample size relative to other studies included (*N* = 6662). All measures of strength (extension, flexion, left and right lateral flexion) were significantly higher in athletes who did not sustain a mTBI compared to the injured cohort. They state that for every pound (0.45 kg) increase in overall neck strength (averaged across the four directions), the odds of sustaining a mTBI was reduced by 5%. Neck strength did not seem to have the same protective effect when field-based impacts were examined over 10 G and 20 G, with two investigations finding that players with stronger necks were more likely to sustain more severe head impacts as measured by HITsp [5, 7]. In addition, Schmidt et al. [7] controlled for player type and separated participants into skill or line groups based on player position. Linemen are typically larger and stronger than skill players and have substantially different roles to skill players. Due to this role difference, linemen have greater exposure to head impacts than skill players. They found that linemen with stronger necks had increased odds of receiving moderate (66–106 G) and severe (> 106 G) head impacts compared with mild (10–66 G) head impacts. They suggested that players with stronger necks may perceive less risk from impact situations and have more sporting ability than those with weaker necks, and so are more willing to engage in high-energy collisions. They also posited that anticipation and being the striking player may play a role, as the same relationship was not found in skill players, who are not as responsible for initiation of contact as expected by linemen. Another finding was that skill players who could activate neck musculature faster had increased odds of sustaining severe impacts. The investigators suggested that by acting faster to mitigate HIK, the forces imparted on the head act over a shorter amount of time and so are more severe. Baker et al. [8] investigated pre-season neck endurance as opposed to MVIC, measured by the deep neck flexor endurance test (DNFET, laying supine and holding a small neck flexion for as long as possible). While athletes in their study who sustained a concussion had lower pre-season DNFET scores, no significant group differences could be found. Interestingly though, they did find a moderate correlation of DNFET improvement with mTBI recovery, which has some potential clinical utility.

### Neck Strength in Laboratory-Based Studies

Evidence for the protective capacity of neck strength is also mixed when considering lower, controlled impacts in a laboratory setting. Eckner et al. [6] found significant main effects for neck strength on both linear and rotational head velocity. Although their study included children, the effect remained when adjusting for age as a covariate in the model. They also found rate of force development to be a significant predictor of linear and rotational head velocity mitigation in all directions except right lateral flexion. Tierney et al. [9] also found larger values of neck strength significantly reduced angular head acceleration and displacement from the perturbation when comparing between sexes. Both of these studies assessed forced neck flexion and extension. Alsalaheen et al. [11] also compared results between the sexes but only evaluated forced neck extension. Despite the men in their study having stronger necks, no significant differences were seen between males and females for angular velocity or displacement. Bretzin et al. [14] also found a relationship between neck muscle strength and HIK during football/soccer heading. They found that three of the six (flexion, left lateral flexion and left rotation) directions of neck strength they measured correlated significantly with linear acceleration at a low ball-speed, and four of six (flexion, left and right lateral flexion, and left rotation) at a high ball-speed. They did not report if players were standing, running or jumping, or where they were aiming the ball, but did control ball-speed. Gutierrez et al. [16], on the other hand, did not control ball-speed (using a throw-in from another football/soccer player to closely mimic real-life situations), but did report on player motion (standing only) and had players aim left, right or forward. They found neck strength moderately and consistently negatively correlated with linear head accelerations, for all directions of strength they measured (flexion, extension, left and right lateral flexion) and all football/soccer heading directions. Dezman et al. [12], while not finding a protective effect from neck strength magnitude alone, did find that athletes with more symmetrical flexion and extension strength experienced lower angular head acceleration during football/soccer heading than those with greater strength imbalance. Teymouri et al. [17] found no significant correlation between neck flexion strength and force exerted by the ball on the head. Tierney et al. [18] found no correlation between neck strength and head acceleration during heading in a control condition without headgear but did find significant negative correlations between these variables for two headgear conditions. The neck-strengthening program used by Becker et al. [13] did not significantly increase strength for the two intervention groups as compared to a control group. Subsequently, football/soccer heading HIK were also not reduced for the intervention groups or the control group. The intervention group in Müller and Zentgraf [27] had significantly increased neck strength (but not lateral strength symmetry) post-intervention as compared with controls. Compared to pre-intervention measurements, post-intervention mean peak linear head acceleration during heading was reduced by 1.5 G in the low-speed condition and 1.4 G in the high-speed condition; however, only the low-speed condition results reached significance. Morris and Popper [21] found neck strength did not predict head deflection in their impact sled study. However, they did find strong negative correlations between head deflection and headrest force, which is the force exerted on the headrest by a participant the instant before impact. They suggested that motivation plays a key role in the level of force participants are willing to exert. During static neck strength testing, participants are not as motivated to exert maximal neck force as they are during impact sled runs (to protect themselves from the impact). This finding is significant for all studies that assess neck strength included in the current review, as strength was always tested statically in non-motivated conditions. As Morris and Popper [21] found, neck MVIC strength measures taken in the laboratory may not reflect neck force exerted during motivated situations like gameplay and training impacts or other forms of head perturbation. This could contribute to the lack of protective relationship existing between static neck strength and head impact mitigation in Schmidt et al. [7], while such a relationship was found between head impact mitigation in the laboratory and field (when compared to those with lower stiffness, players with higher levels of stiffness in laboratory perturbations had lower odds of sustaining more severe field-based impacts). While the participants had their heads perturbed by a weight drop, they may have been more motivated to maximally activate neck musculature to dissipate the force than in the static neck strength testing condition.

### Head-Neck Size Variables in Field-Based Studies

Another characteristic of interest in some of the included studies is the size of the head and neck. Some researchers propose that neck size can be used as a quick and simple proxy measure of other neck features, such as strength [22]. Studies included in this review did find correlations between head-neck size and mass measurements and neck strength [14, 23]. In addition, increased size of the head-neck segment would reduce head accelerations, whereby for the same force a body with larger mass experiences less acceleration than a body with smaller mass. Indeed, mathematical models have shown a correlation between less head mass and increased accelerations [29]. Collins et al. [10] evaluated neck length and head and neck circumference, as well as the ratio of these anatomical features. Viewing their overall results, neck circumference and neck/head circumference were significantly smaller for the mTBI group. However, when compared within sex, neck circumference difference was not significantly different between groups for either males or females, and neck/head circumference ratio only remained significantly different between the groups for males. Esopenko et al. [22] found neck circumference not to be related to either mTBI sustained during college or to mTBI history. Kelshaw et al. [23] reported head and neck circumference and neck length but did not relate these to HIK in their study, instead relating them to neck strength measurements. A moderately positive correlation was found between neck circumference and extension strength, but no other relationships were statistically significant. Schmidt et al. [7] found that players above the median in neck muscle PCSA were at increased odds of moderate and severe head impacts compared to those below the median PCSA. Again, this could be because players in the upper half of the PCSA distribution feel safer entering high-energy collisions, or due to expectations of these players to participate in collisions.

### Head-Neck Size Variables in Laboratory-Based Studies

In controlled perturbation conditions, head and neck size metrics proved to reduce or partially reduce head deflection severity in some studies [6, 9, 19, 20], but not in others [11]. Alsalaheen et al. [11] found significant differences in neck circumference and SCM PSCA between males and females (males had larger necks), but no difference in angular velocity or displacement. However, it was found that females had a significantly larger neuromuscular response (mean baseline and peak EMG across all perturbation conditions), which may indicate that the males were able to rely on their larger size (and strength) to mitigate impact forces, while females had to use a larger percentage of activation. This is despite the fact that this perturbation protocol was normalised to participant weight. Similarly, Tierney et al. [9] also found that females had faster and larger neuromuscular responses than males. However, in contrast to findings from Alsalaheen et al. [11], Tierney et al. [9] observed significantly greater peak acceleration and head displacement in females compared to males. Tierney et al. [18] found neck circumference and head-neck mass and length to be significantly negatively correlated with resultant head acceleration during headers in both their headgear conditions, but only head-neck mass in their non-headgear condition. Headgear appeared to have different effects on HIK between sexes. Males experienced lower head accelerations when wearing headgear, while females experienced higher head accelerations. The authors attribute this finding to a feeling of safety the headgear might provide and participants feeling as if they must strike the ball harder while wearing headgear. They suggested that the lower head-neck stability (head-neck mass, neck girth, neck flexion and extension strength) in females compared to males in their study may account for why this was not seen in males. Teymouri et al. [17] found head circumference to significantly negatively correlate with the amount of force measured on the head during headers. What this result tells us about injury risk prevention is unclear, however, as it has been reported that injuries during headers depend more on head acceleration than the applied force [30]. Bretzin et al. [14] found neck girth to be significantly and negatively associated with HIK, more so as ball speed increased. Despite measuring head-neck length and mass, they did not report if these correlated with HIK.

### Differences Between Females and Males

Several of the studies included in the present review performed a group analysis between males and females. The general trend in such studies is stronger and larger necks in males as compared to females. This trend, at times, has been taken as evidence that having a stronger and larger neck is responsible for any measured benefits in head impact mitigation. However, as previously discussed, Alsalaheen et al. [11] did find that males and females utilised different neuromuscular strategies under the same load. Tierney et al. [9] also found head acceleration and angular displacement of males to be significantly lower than that of females in their controlled perturbation experiment. When males had knowledge of the incoming perturbation, their angular acceleration was 25% less than without this knowledge. No significant difference was found between the known and unknown conditions in females. While Williams et al. [28] found no significant difference in peak linear head acceleration between males and females, they did see a marked difference in response to impacts, where over 50% of impacts sustained by females resulted in uncontrolled whiplash while only one such event occurred in their male cohort. They attributed this to decreased stability in the female cervical spine as compared to males. As men and women use different strategies to stabilise the head [11], physical size and strength differences alone may not be the only factors at play. In future studies, comparisons should be performed within groups in addition to between them to minimise the influence of any different neuromuscular strategies employed by the sexes on findings.

### Reviews From Other Perspectives

It is worth noting that the role of the head and neck in mTBI risk has been examined by several other reviews through other perspectives. The review by Elliott et al. [31] evaluated the relationship between neck strength and head and neck injuries in sport, and whether neck exercise interventions reduce these injuries. Their review shares similar aims with this review but is broader in its inclusion of head and neck injuries other than solely mTBI, addresses strengthening interventions specifically, and is narrower in its focus on only athletic populations. Other reviews on this topic have either not been systematic [32–41] or have had a more narrow scope than this review by limiting their search to either specific experimental paradigms [42, 43] or specific physical neck characteristics [44]. To our knowledge, this review is the first systematic review to holistically assess physical characteristics of the head and neck and how they relate to mTBI risk in a broad range of settings. We also included military cohorts in our review, which further differentiates it from the existing literature. In our search terms, we listed popular sports which are known to have high rates of mTBI. However, studies on sports other than those included in our search terms were also captured in our search with the inclusion of “athlete” and “sport” keywords and subject headings, as well as by our manual search efforts of reference lists.

It should be noted that only papers written in English were screened, which is a limitation of this review. Participant age was restricted to 13–65 years, which excluded papers that observed participants outside of this age threshold. Occupational (excluding military service) and vehicular mTBI studies were not included in the search strategy. Additionally, only three of the included studies directly assessed mTBI incidence [8, 10, 22]. The other 19 included studies used indirect measures of mTBI risk, which may limit applicability of any conclusions drawn.

## Conclusion

From the studies included in the current review and the broader literature, there appears to be evidence of some interaction between physical characteristics of the head-neck segment and mTBI risk, in terms of incidence and biomechanical response to impact. However, these characteristics alone appear to not be sufficient to wholly predict mTBI risk. Other factors, such as neuromuscular response, behaviour, sex, and anticipation of impact also contribute to head impact mitigation. Further large-scale robust prospective investigations are required to determine the exact role that physical head-neck characteristics play in mTBI prevention and impact mitigation.

## Footnotes

1 Bretzin et al. [14] list incorrect units of rad/s^2^ for rotational velocity.

2 Bretzin et al. [14] list incorrect units of m/s for linear acceleration.

## Declarations

### Data availability

All data generated or analysed during this study are included in this published article or the studies it cites.
